# Mortality and trends of pulmonary arterial hypertension, 1990–2021: a population-based study

**DOI:** 10.3389/fcvm.2025.1617610

**Published:** 2025-09-03

**Authors:** Linhong Jiang, Xinliao Deng, Yuchen He, Yu Ai, Hui Li, Peijun Li, Xiaodan Liu

**Affiliations:** ^1^ School of Rehabilitation Science, Shanghai University of Traditional Chinese Medicine, Shanghai, China; ^2^ Engineering Research Center of Traditional Chinese Medicine Intelligent Rehabilitation, Ministry of Education, Shanghai, China; ^3^ Shanghai Academy of Traditional Chinese Medicine, Institute of Rehabilitation Medicine, Shanghai, China

**Keywords:** hypertension, pulmonary arterial hypertension, global burden of disease, trends, mortality

## Abstract

**Background:**

Pulmonary arterial hypertension (PAH) is a progressive disease associated with high fatality rate. Comprehensive evidence concerning the epidemiology of PAH mortality is lacking. The present study aimed to assess the mortality and trends of PAH at global, regional, and national levels from 1990 to 2021.

**Methods:**

The estimates and their 95% uncertainty interval for case number of PAH were collected from the Global Burden of Disease, Injuries, and Risk Factors Study (GBD) 2021. Age standardization by direct method was used to estimate the age-standardized mortality rate (ASMR) for PAH. We investigated the temporal trends of estimated ASMR from 1990 to 2021 and further predicted its changes in the next 15 years, decomposed the trends based on demographic factors and epidemiological changes, and quantified the cross-country health inequalities.

**Results:**

The global ASMR of PAH decreased from 0.35 (per 100,000 population) in 1990 to 0.27 (per 100,000 population) in 2021, with an annual percentage change of −0.82, while number of deaths increased from 14,842 in 1990 to 22,021 in 2021, an increase of 48.37%. Population growth and aging were the major drivers contributing to the number of deaths, accounting for 93.88% and 32.26%. Of note, there were significant health inequalities across 204 countries and territories, with the slope index of inequality rising over time. Projection of the global burden of PAH from 2022 to 2036 demonstrated a progressive increase in case number, but the ASMR remained largely unchanged.

**Conclusion:**

PAH remains a major public health concern in some regions, particularly in high sociodemographic index region. Adequate diagnosis and targeted treatment of PAH are urgently needed to achieve a reduction in mortality.

## Background

Pulmonary arterial hypertension (PAH) is a rare condition characterized by progressive occlusion of small pulmonary arteries, leading to a gradual increase in pulmonary vascular resistance, right ventricular failure, and ultimately death (1, 2). Although significant advances in the understanding of the underlying mechanisms and development of many targeted therapies, PAH remains a challenging condition with high fatality rate (3), therefore, a comprehensive elucidation in the epidemiology of PAH is of great significance. However, in recent years, investigations of PAH mortality have been performed in very limited geographic areas, mostly in specific countries, using data from national health systems, and the lack of an age-standardization process greatly limits cross-region and cross-country comparisons (4). Certainly, a global estimation of PAH mortality can fill in the lack of disease statistics, and a comprehensive analysis of its changing trends can help to better understand disease epidemiology and optimize the allocation of health resources, therefore contributing to reducing the future burden of disease.

The Global Burden of Disease, Injuries, and Risk Factors Study (GBD) 2021 provides an up-to-date, comprehensive set of data on the fatal burden of disease, summarizing 288 cause-specific mortality metrics by age and sex for 204 countries and territories for the period 1990–2021, and is an update of previously published estimates for 1990–2019 (5). In the current study, we used the GBD 2021 data to (1) describe the trends in PAH mortality at the global, regional, and national levels, (2) determine the contribution of population size, age structure, and epidemiologic changes to PAH mortality, (3) analyze cross-country inequalities, and (4) forecast the changes to 2036. Significantly, our findings can serve as a valuable extension and complement to the previous study (6) and contribute to the development of country-specific PAH public health strategies.

## Methods

### Data acquisition and download

The GBD 2021 provided an in-depth assessment of health detriments associated with 371 diseases, injuries, and impairments and 88 risk factors across 204 countries and territories, using the latest epidemiological data and improved standardized methodologies (7). In the present study, the determinations and their 95% uncertainty interval (UI) for mortality relating to PAH were drawn from the GBD 2021 at global, regional, and national levels. Meanwhile, the study employed the sociodemographic index (SDI), a measure that quantifies a region's sociodemographic progression based on income, education, and fertility circumstances (8), which was also used.

The number of deaths and age-standardized rate (ASR) of PAH from 1990 to 2021, by sex, region, and country, were extracted from the Global Health Data Exchange query tool (https://vizhub.healthdata.org/gbd-results/). Specifically, we chose “Cause” as “Pulmonary Arterial Hypertension”, “Measure” as “Deaths”, “Metric” as “Number” and “Rate” to obtain the mortality data across 204 countries and territories all over the world, with a search period from 1990 to 2021. Related forecast statistics on the world population from 2020 to 2036 were sourced from the GBD database (https://www.ghdx.healthdata.org/record/ihme-data/global-population-forecasts-2017-2100).

### Joinpoint regression analysis

ASR and average annual percentage change (AAPC) were used for temporal trends in PAH mortality. AAPC values represent annual percent change (increase, decrease, or no change). Statistically, an upward trend is indicated if the lower 95% confidence interval (CI) of the AAPC estimate exceeds zero. In contrast, a downward trend is indicated if the upper 95% CI of the AAPC estimate is less than zero. When the 95% CI of the AAPC includes zero, the trend remains stable (9). The significance level was set at *P* < 0.05.

### Decomposition analysis

To gain a better understanding of explanatory factors that drove change in PAH mortality between 1990 and 2021, we performed a decomposition analysis of the case number by population size, age structure, and epidemiologic changes. Epidemiological changes refer to the underlying age and population-adjusted mortality and morbidity rates (10).

### Cross-country inequalities analysis

The total mortality rate was extracted for inequality analysis. Slope of inequality index (SII) and the concentration index (CI) were used to assess absolute and relative income-related inequalities across countries (10, 11). The SII was calculated by regression of the country-level mortality due to PAH in all age populations on the sociodemographic development-related relative position scale, defined by the midpoint of the cumulative class range of the population ranked by SDI. The CI is utilized to assess the relative disparity in the burden of PAH among countries by fitting the Lorenz concentration curve based on cumulative mortality and cumulative population (12).

### Projection analysis

Autoregressive integrated moving average (ARIMA) is a widely used time series analysis method for forecasting future values. In the present study, we built the ARIMA model to forecast the number of deaths and age-standardized mortality rate (ASMR) for PAH from 2022 to 2036. Importantly, this study used the number of deaths and the ASMR from 1990 to 2021 as the base of prediction model. In brief, the ARIMA model based on GBD 2021 used differential processing until the sequence was smooth. The “auto.arima ().” function for selecting the best-optimized model depends on the Akaike information criterion. The autocorrelation function (ACF) and the partial autocorrelation function (PACF) were used to judge the appropriate model parameters, while the Ljung–Box *Q*-test, ACF, and PACF of residuals were utilized to determine whether the residuals of the optimal model meet the requirements of white noise sequences (13, 14). The ARIMA model was constructed using the “forecast” package of R.

### Statistics analysis

The mortality rate was expressed as the estimate per 100,000 population and its 95% UI. All statistical analysis and visualization were executed using R software (version 4.3.2).

## Results

### Global, regional, and national trends in PAH mortality

Globally, the case number of PAH increased from 14,842 (95% UI: 12,370–17,485) in 1990 to 22,021 (95% UI: 18,239–25,352) in 2021, an increase of 48.37%. The ASMR for PAH was 0.35 (95% UI: 0.29–0.42) in 1990, 0.27 (95% UI: 0.23–0.32) in 2021, and −0.82 (95% CI: −0.95 to −0.68) in AAPC, demonstrating a downward trend. In both 1990 and 2021, the case number of PAH was slightly higher in females, and the ASMR was higher in females in 2021. In terms of 5 SDI regions, there was an increasing trend in the number of PAH mortality, with the highest increase observed in the high SDI region, an increase of 76.58%. The decreasing trend in ASMR was observed in all SDI regions, additionally, a largely decreased trend of the change for PAH ASMR was demonstrated in the high-middle SDI region.

**Figure 1:**
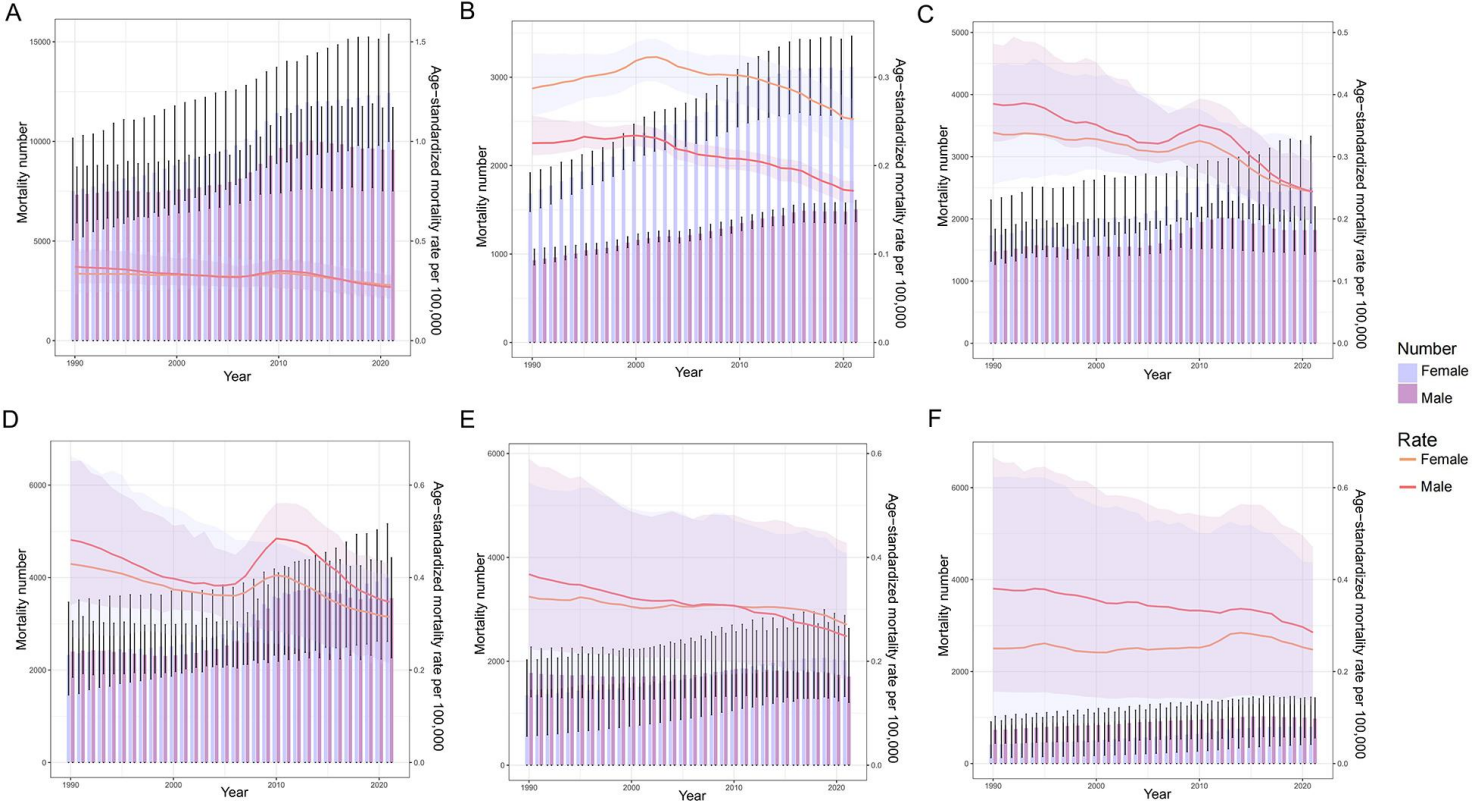
Trend in mortality number and ASMR of PAH. **(A)** The global trend in mortality number and ASMR of PAH from 1990 to 2021; **(B)** The trend in mortality number and ASMR of PAH in the high SDI region from 1990 to 2021; **(C)** The trend in mortality number and ASMR of PAH in the high-middle SDI region from 1990 to 2021; **(D)** The trend in mortality number and ASMR of PAH in the middle SDI region from 1990 to 2021; **(E)** The trend in mortality number and ASMR of PAH in the low-middle SDI region from 1990 to 2021; **(F)** The trend in mortality number and ASMR of PAH in the low SDI region from 1990 to 2021. ASMR, age-standardized mortality rate; PAH, pulmonary arterial hypertension; SDI, sociodemographic index.

**Figure 2:**
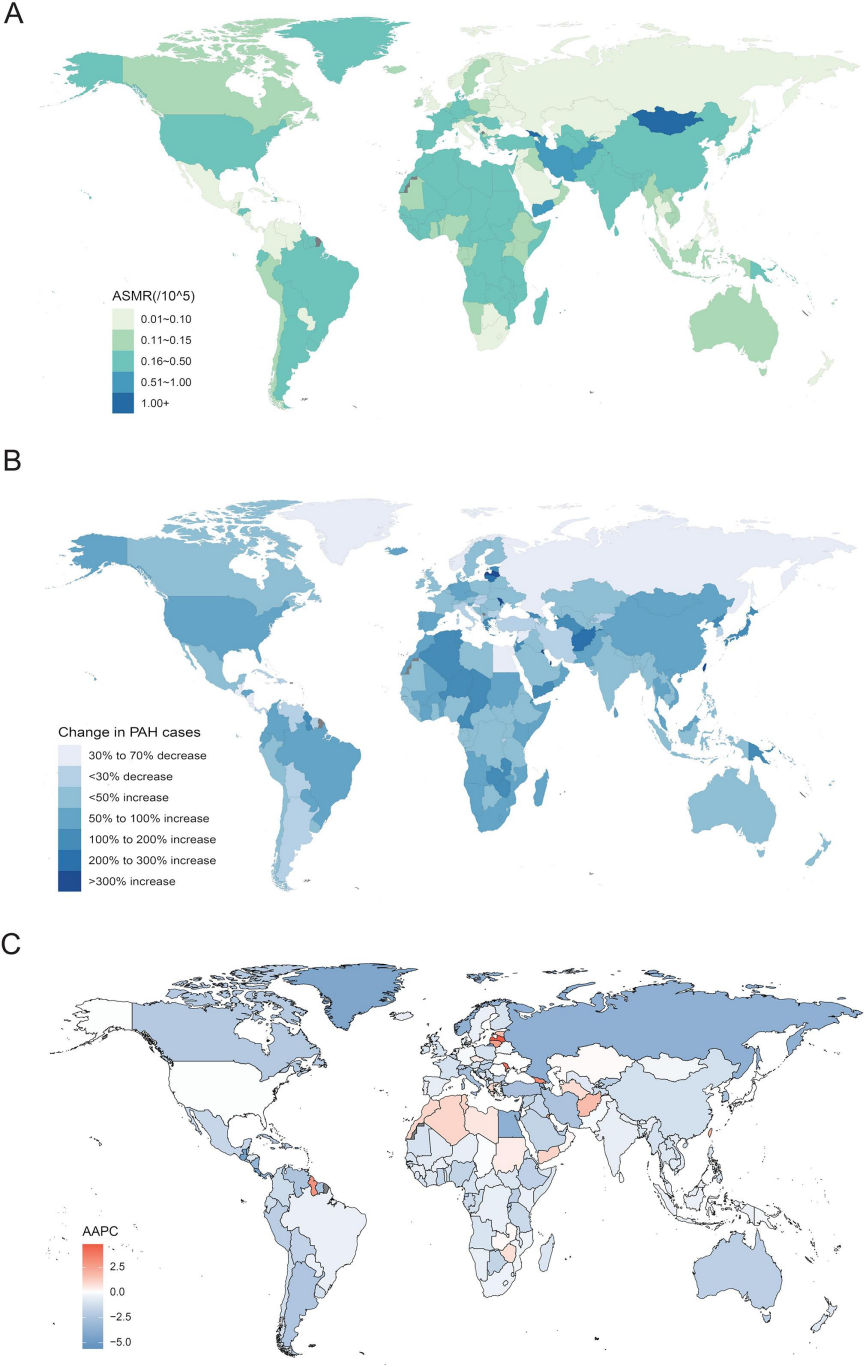
ASMR, relative change in case number of mortality and AAPC of PAH. **(A)** The ASMR of PAH in 2021; **(B)** The relative change in case number of mortality of PAH from 1990 to 2021; **(C)** The AAPC of ASMR of PAH from 1990 to 2021. ASMR, age-standardized mortality rate; PAH, pulmonary arterial hypertension; AAPC, average annual percentage change.

At the regional level, the largely decreased trend of estimated ASMR occurred in Eastern Europe, followed by Southern Latin America and Central Latin America. However, an upward trend in the PAH ASMR is Central Asia (Table 1 and Figure 1). At the national level, the PAH ASMR varies considerably across the world, with the highest ASMR observed in Mongolia, followed by Georgia and Tajikistan. The mortality cases of PAH were higher in most countries in 2021 than in 1990, and the highest mortality cases were recorded in China in 2021, followed by India and the United States of America. The most pronounced increase was observed in Taiwan (Province of China), an increase of 509.68%. Encouragingly, in 179 countries of the world, AAPC was less than zero, indicating a downward trend of ASMR, and the top three countries were Puerto Rico, Guatemala, and Costa Rica. Only 25 countries reported an increasing ASMR between 1990 and 2021, and the largest increase in ASMR was observed in Latvia at 4.71, followed by the Republic of Moldova and Mauritius (Figure 2 and Supplementary Table S1).

**Table 1:** The case number and age-standardized mortality rate of pulmonary arterial hypertension in 1990 and 2021, and its temporal trends from 1990 to 2021.

Location	1990		2021			1990–2021
	Case number (95% UI)	ASMR (95% UI)	Case number (95% UI)	ASMR (95% UI)		AAPC (95% CI)
Global	14,842 (12,370, 17,485)	0.35 (0.29, 0.42)	22,021 (18,239, 25,352)	0.27 (0.23, 0.32)		−0.82 (−0.95, −0.68)
Sex						
Male	7,326 (5,917, 8,715)	0.37 (0.29, 0.46)	9,579 (7,516, 11,703)	0.27 (0.21, 0.33)		−1.07 (−1.27, −0.87)
Female	7,516 (5,058, 10,161)	0.34 (0.23, 0.45)	12,442 (10,001, 15,376)	0.28 (0.22, 0.34)		−0.61 (−0.73, −0.49)
SDI						
High SDI	2,617 (2,379, 2,850)	0.26 (0.24, 0.28)	4,621 (3,919, 5,054)	0.22 (0.19, 0.23)		−0.59 (−0.75, −0.44)
High-middle SDI	3,214 (2,772, 3,908)	0.35 (0.31, 0.43)	4,326 (3,594, 5,141)	0.24 (0.20, 0.29)		−1.22 (−1.50, −0.95)
Middle SDI	4,729 (3,774, 5,852)	0.45 (0.35, 0.58)	7,548 (5,141, 9,026)	0.33 (0.22, 0.39)		−1.10 (−1.31, −0.88)
Low-middle SDI	3,125 (2,220, 3,904)	0.35 (0.22, 0.47)	3,728 (2,757, 5,091)	0.26 (0.18, 0.38)		−0.89 (−1.03, −0.75)
Low SDI	1,145 (740, 1,755)	0.32 (0.15, 0.51)	1,782 (1,147, 2,544)	0.27 (0.15, 0.4)		−0.53 (−0.81, −0.25)*
Region						
Andean Latin America	84 (56, 112)	0.28 (0.21, 0.35)	91 (72,119)	0.16 (0.12,0.2)	−1.83 (−2.14, −1.51)	
Australasia	45 (39, 57)	0.21 (0.18, 0.26)	58 (49, 65)	0.11 (0.10, 0.13)	−1.85 (−2.22, −1.49)	
Caribbean	124 (83, 169)	0.38 (0.28, 0.49)	95 (66, 130)	0.20 (0.13, 0.29)	−1.96 (−2.19, −1.73)	
Central Asia	208 (163, 240)	0.40 (0.31, 0.47)	319 (261, 382)	0.41 (0.34, 0.48)	0.11 (−0.18, 0.40)	
Central Europe	355 (309, 394)	0.26 (0.22, 0.28)	438 (398, 479)	0.21 (0.19, 0.23)	−0.70 (−0.96, −0.43)	
Central Latin America	180 (157, 210)	0.16 (0.14, 0.19)	201 (177, 230)	0.08 (0.07, 0.1)	−2.13 (−2.36, −1.90)	
Central Sub-Saharan Africa	87 (55, 163)	0.24 (0.11, 0.47)	131 (62, 237)	0.19 (0.08, 0.37)	−0.76 (−0.80, −0.73)	
East Asia	4,115 (3,141, 552)	0.59 (0.45, 0.81)	7,490 (4,986, 9,266)	0.41 (0.28, 0.5)	−1.23 (−1.49, −0.96)	
Eastern Europe	563 (512, 651)	0.24 (0.22, 0.27)	278 (258, 300)	0.09 (0.08, 0.1)	−2.98 (−3.74, −2.21)	
Eastern Sub-Saharan Africa	366 (217, 687)	0.27 (0.12, 0.52)	468 (219, 878)	0.18 (0.07, 0.34)	−1.37 (−1.44, −1.29)	
High-income Asia Pacific	434 (410, 459)	0.26 (0.24, 0.27)	1,049 (826, 1,201)	0.23 (0.20, 0.26)	−0.34 (−0.61, −0.06)*	
High-income North America	1,064 (947, 1,167)	0.32 (0.28, 0.35)	1,880 (1,620, 2,043)	0.29 (0.26, 0.31)	−0.26 (−0.43, −0.09)*	
North Africa and Middle East	2,142 (1,309, 273)	0.77 (0.56, 1)	1,896 (1,328, 2,305)	0.44 (0.31, 0.53)	−1.75 (−1.90, −1.61)	
Oceania	12 (8, 19)	0.28 (0.18, 0.53)	25 (17, 43)	0.24 (0.16, 0.48)	−0.47 (−0.55, −0.38)	
South Asia	2,385 (1,502, 341)	0.31 (0.17, 0.5)	3,549 (2,321, 5,532)	0.25 (0.16, 0.42)	−0.63 (−0.88, −0.39)	
Southeast Asia	506 (340, 1,094)	0.15 (0.09, 0.43)	741 (525, 1,850)	0.12 (0.08, 0.32)	−0.76 (−0.79, −0.72)	
Southern Latin America	169 (151, 186)	0.36 (0.32, 0.4)	150 (138, 162)	0.18 (0.17, 0.2)	−2.17 (−2.47, −1.87)	
Southern Sub-Saharan Africa	43 (32, 58)	0.12 (0.08, 0.17)	72 (53, 86)	0.11 (0.08, 0.13)	−0.26 (−0.40, −0.12)*	
Tropical Latin America	394 (373, 412)	0.37 (0.35, 0.39)	779 (714,822)	0.32 (0.29, 0.34)	−0.50 (−0.78, −0.23)*	
Western Europe	1,233 (1,094, 1,380)	0.24 (0.21, 0.27)	1,788 (1,533, 1,943)	0.18 (0.16, 0.19)	−0.94 (−1.19, −0.68)	
Western Sub-Saharan Africa	335 (195, 616)	0.25 (0.09, 0.51)	523 (306, 774)	0.17 (0.07, 0.28)	−1.29 (−1.35, −1.23)	

ASMR, age-standardized mortality rate; UI, uncertainty interval; SDI, sociodemographic index; CI, confidence interval; AAPC, average annual percentage change.

* Indicates *P*-value less than 0.05.

### Drivers of PAH epidemiology: population growth, aging, and epidemiologic changes

**Figure 3:**
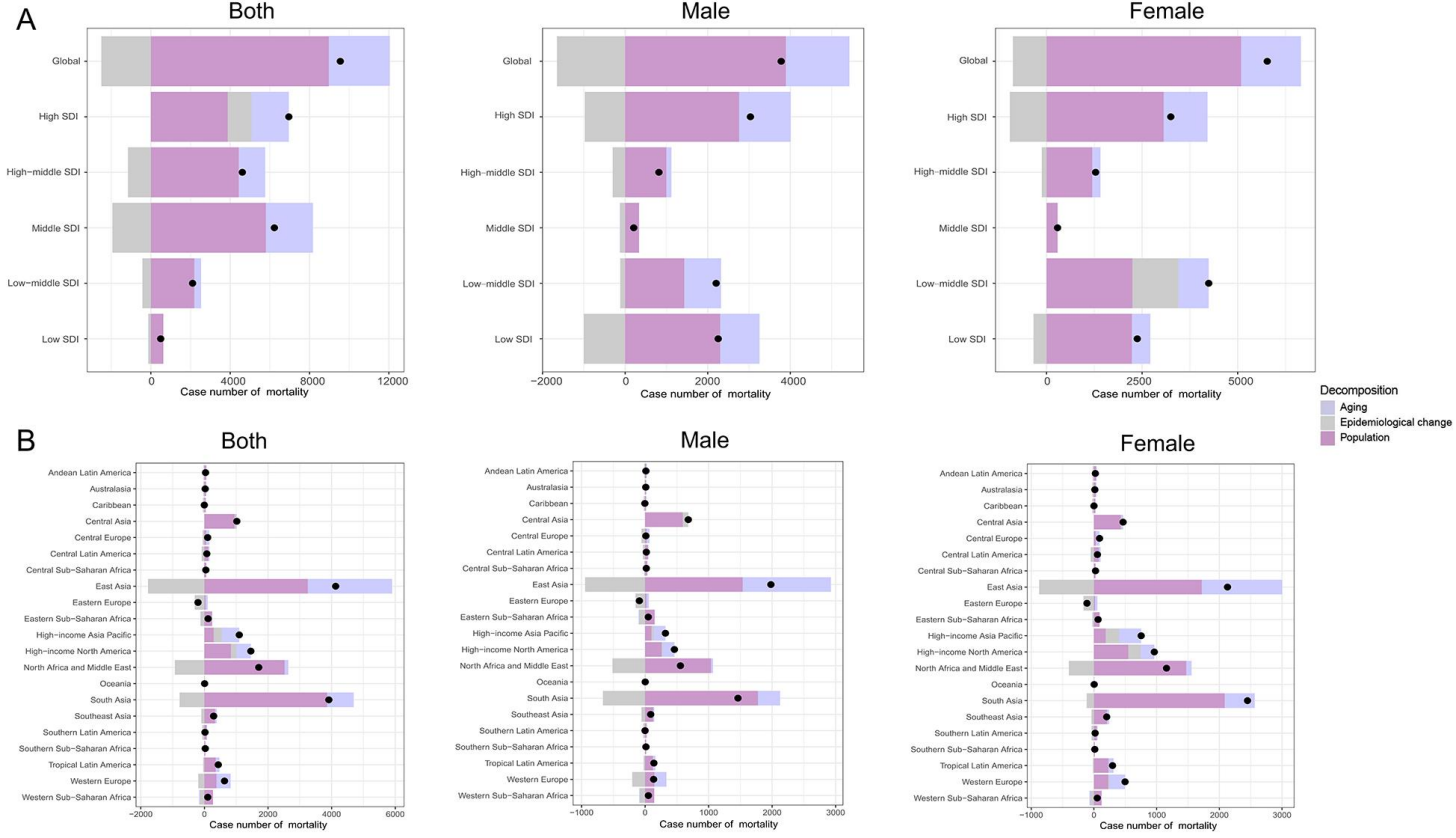
Changes in mortality of PAH according to population-level determinants including aging, population growth and epidemiological change from 1990 to 2021. **(A)** The global and SDI levels by sexes; **(B)** The regions levels by sexes. PAH, pulmonary arterial hypertension; SDI, sociodemographic index.

Over the past 31 years, there was a significant increase in PAH mortality globally and in each SDI region, with the largest increase occurring in the high SDI region. Globally, population and aging contributed 93.88% and 32.26%, respectively, to the increased burden of PAH mortality between 1990 and 2021. The contribution of aging to overall mortality was most pronounced in the middle SDI (38.14%), and decreased where it was 28.94% in the high-middle SDI, 27.05% in the high SDI, 16.09% in the low-middle SDI, and negative growth in the low SDI (−2.50%). Most notably, the mortality of PAH is driven primarily by population growth in the 5 SDI regions with 55.69%, 96.04%, 93.07%, 104.08%, and 126.62%, respectively. The epidemiological changes in age-adjusted and population-adjusted PAH have decreased globally, and the decrease was more evident in the middle SDI (−31.20%) but increased in the high SDI (17.25%). In terms of GBD regions, aging and population growth were major drivers of change in PAH mortality in most regions, additionally, the contribution of aging and population growth to overall mortality was most pronounced in Southern Latin America with 88.51% and 337.55%. Noticeably, most GBD regions revealed a decrease in underlying epidemiologic changes, however, 5 GBD regions demonstrated an increase in epidemiologic changes, including the Caribbean (1,869.17%), Eastern Europe (152.11%), High-income Asia Pacific (22.96%), High-income North America (11.89%), and Central Asia (4.74%). The above results are presented in Table 2 and Figure 3.

**Table 2:** Changes in mortality number according to population-level determinants from 1990 to 2021 at the global and regional level.

Location	Overall difference	Change due to population-level determinants (% contribution to the total changes)		
		Aging	Population	Epidemiological change
Global	9,552.19	3,081.95 (32.26%)	8,967.39 (93.88%)	−2,497.14 (−26.14%)
SDI				
High SDI	6,955.18	1,881.56 (27.05%)	3,873.61 (55.69%)	1,200.01 (17.25%)
High-middle SDI	4,612.42	1,334.94 (28.94%)	4,429.77 (96.04%)	−1,152.29 (−24.98%)
Middle SDI	6,226.71	2,374.64 (38.14%)	5,795.05 (93.07%)	−1,942.98 (−31.20%)
Low-middle SDI	2,104.78	338.60 (16.09%)	2,190.68 (104.08%)	−424.50 (−20.17%)
Low SDI	496.09	−12.39 (−2.50%)	628.14 (126.62%)	−119.66 (−24.12%)
Region				
Andean Latin America	36.48	12.98 (35.57%)	54.23 (148.65%)	−30.73 (−84.23%)
Australasia	25.76	20.64 (80.11%)	40.95 (158.97%)	−35.82 (−139.07%)
Caribbean	−2.53	7.95 (−314.19%)	36.81 (−1,454.94%)	−47.29 (1,869.17%)
Central Asia	1,019.36	23.63 (2.32%)	947.41 (92.94%)	48.31 (4.74%)
Central Europe	101.90	102.81 (100.89%)	53.57 (52.57%)	−54.47 (−53.45%)
Central Latin America	75.51	38.82 (51.41%)	120.06 (159.00%)	−83.38 (−110.42%)
Central Sub-Saharan Africa	45.70	−3.75 (−8.20%)	59.51 (130.21%)	−10.06 (−22.02%)
East Asia	4,126.50	2,647.79 (64.17%)	3,251.80 (78.80%)	−1,773.09 (−42.97%)
Eastern Europe	−200.57	67.70 (−33.76%)	36.82 (−18.36%)	−305.10 (152.11%)
Eastern Sub-Saharan Africa	116.23	−15.11 (−13.00%)	242.84 (208.93%)	−111.50 (−95.93%)
High-income Asia Pacific	1,095.75	552.01 (50.38%)	292.19 (26.67%)	251.55 (22.96%)
High-income North America	1,459.07	451.39 (30.94%)	834.14 (57.17%)	173.54 (11.89%)
North Africa and Middle East	1,708.63	115.37 (6.75%)	2,523.00 (147.66%)	−929.74 (−54.41%)
Oceania	6.73	0.32 (4.70%)	8.36 (124.16%)	−1.94 (−28.87%)
South Asia	3,912.40	835.21 (21.35%)	3,857.67 (98.60%)	−780.49 (−19.95%)
Southeast Asia	291.35	54.00 (18.53%)	334.80 (114.91%)	−97.44 (−33.44%)
Southern Latin America	19.68	17.42 (88.51%)	66.43 (337.55%)	−64.16 (−326.04%)
Southern Sub-Saharan Africa	27.58	0.43 (1.55%)	28.89 (104.74%)	−1.74 (−6.30%)
Tropical Latin America	435.38	119.41 (27.43%)	353.71 (81.24%)	−37.73 (−8.67%)
Western Europe	630.98	443.60 (70.30%)	380.78 (60.35%)	−193.40 (−30.65%)
Western Sub-Saharan Africa	106.06	−31.98 (−30.16%)	271.45 (255.94%)	−133.41 (−125.79%)

SDI, sociodemographic index.

### Cross-national PAH health inequality

**Figure 4:**
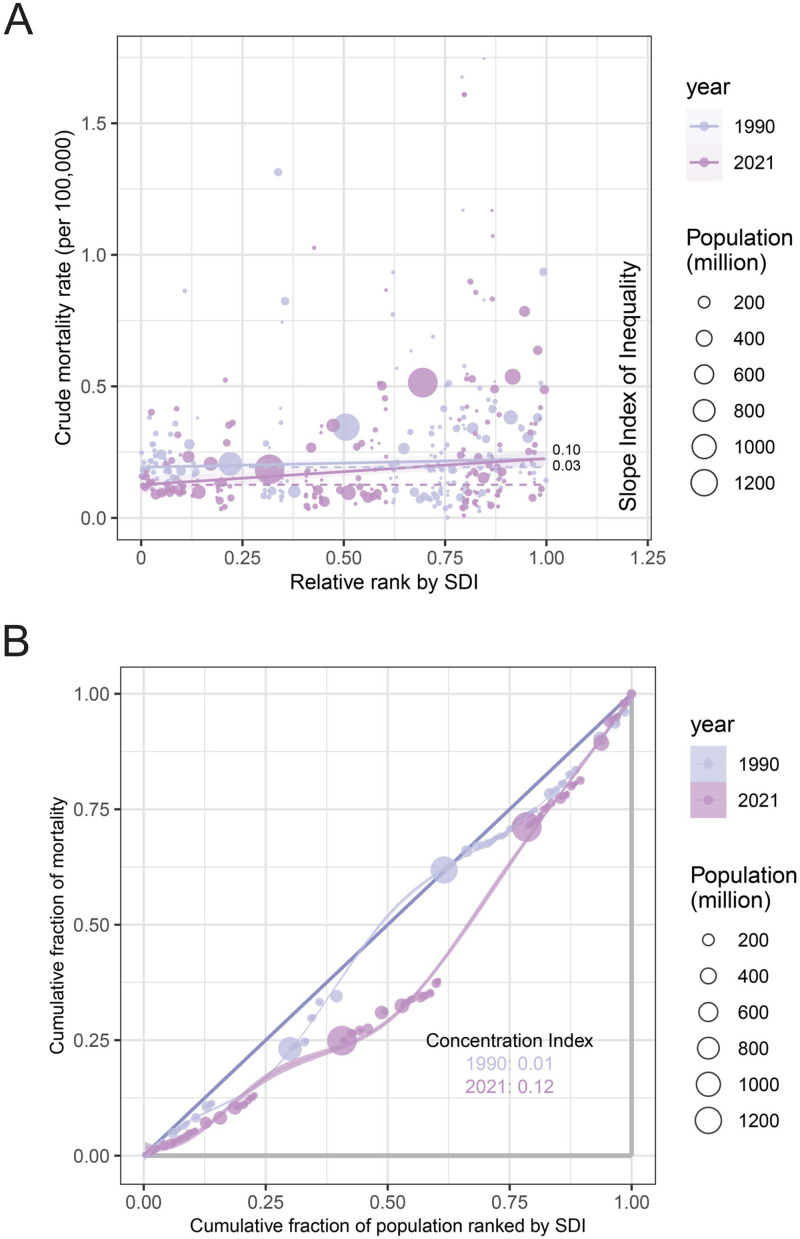
Cross-national PAH health inequality. **(A)** Health inequality regression curves for the mortality of PAH from 1990 to 2021 across the world; **(B)** Concentration curves for the mortality of PAH from 1990 to 2021 across the world. PAH, pulmonary arterial hypertension; SDI, sociodemographic index.

Notable absolute and relative inequalities associated with SDI were observed for PAH burden, with these metrics increasing significantly over time. Importantly, it suggested that higher mortality is disproportionately concentrated in countries with higher SDI. In 1990 and 2021, the SII for PAH mortality was 0.03 and 0.10 respectively, implicating there was an excess of 0.03 (per 100,000 population) mortality in the country with the highest SDI compared to that in the country with the lowest SDI in 1990, and this gap further widened to 0.10 (per 100,000 population) in 2021. Moreover, the CI for PAH mortality also presented an increasing trend from 1990 to 2021 (Figure 4).

### Predicted trends

**Figure 5:**
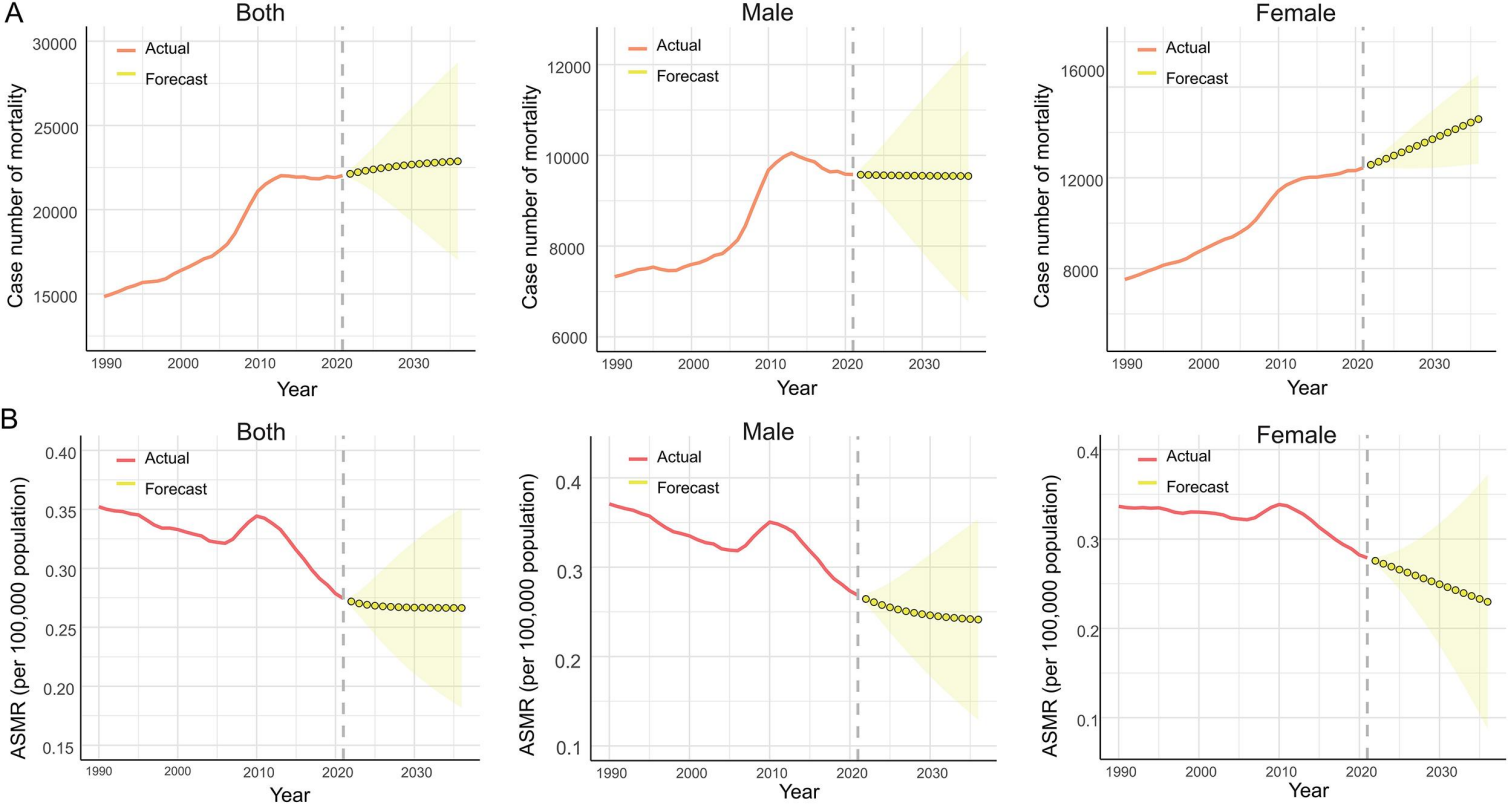
Projected trends of PAH from 2022 to 2036. **(A)** The global change trends of case number of mortality of PAH from 1990 to 2021, and its predicted trends between 2022 and 2036 by sexes; **(B)** The global change trends of ASMR of PAH from 1990 to 2021, and its predicted trends between 2022 and 2036 by sexes. PAH, pulmonary arterial hypertension; ASMR, age-standardized mortality rate.

To predict trends in the global ASMR and case number of mortality by sexes from 2022 to 2036, the ARIMA model was constructed using on the number of death and the ASMR from 1990 to 2021. The predicted mortality number kept growing over the next 15 years, increasing from 22,127 in 2022 to 22,873 in 2,036. Notably, the ASMR for PAH was projected to trend essentially unchanged after 2021, with an estimate of 0.27 (per 100,000 population) in 2036. The results were delineated in Figure 5, Supplementary Tables S2, S3.

## Discussion

Over the past decades, PAH has received heightened attention from clinicians for its high fatality rate and poor long-term outcomes (15). To the best of our knowledge, the present study is first to provide the most up-to-date data on the mortality of PAH at global, regional, and national levels from 1990 to 2021, and further present a comprehensive evaluation based on trend, decomposition, inequality, and predictive analyses.

Globally, we observed a decreasing trend in the ASMR attributable to PAH from 1990 to 2021, indicating significant advances in the long-term management and therapy of PAH over the last decades. This change closely aligns with the modern PAH targeted therapies that were approved and gradually became widespread between 1990 and 2021.

During this period, targeted drugs such as endothelin receptor antagonists (bosentan in 2001 and ambrisentan in 2007), phosphodiesterase-5 inhibitors (sildenafil in 2005), prostacyclin analogues, and receptor agonists (iloprost in 2004, treprostinil in 2002, and selexipag in 2015) were successively launched and incorporated into international guidelines for PAH (16–18), significantly improving functional capacity and hemodynamics and reducing hospital admissions. In addition to targeted drug therapy, surgical treatment (including pulmonary endarterectomy, balloon pulmonary angioplasty, and lung transplantation etc.) and updates to comprehensive management concepts have also significantly improved the survival rate of PAH patients (2, 19). However, the global death cases due to PAH increased from 14,842 in 1990 to 22,021 in 2022, reflecting the impact of global population growth and aging. Additionally, in 2021, we noted a significantly increased overall disease burden from PAH among females compared with males. Evidence suggests that sex is a major disease modifier for PAH, and sex hormones play key roles in the pathophysiology of this disease (20). However, this sex inequality in PAH is not only attributable to physiological differences in sex hormones between the sexes but may also be attributable to lifestyle, socio-economic factors, and cultural factors. Specifically, first, occupational and household air-pollution exposure is sex-skewed. Women in low- and middle-income countries spend more time cooking with solid fuels and therefore have higher PM 2.5 exposure, a recognized risk factor for PAH (21). Second, economic barriers are gendered. In 71% of countries women have lower health-insurance coverage or face higher out-of-pocket costs for specialist referral, reducing access to definitive diagnosis (22). Finally, cultural restrictions in some regions limit women's mobility, preventing attendance at tertiary centres that perform catheterization (23). Therefore, there is a need to further consider sex-specific treatment strategies to improve treatment outcomes and to make treatment programs more targeted.

At the SDI level, the results suggested that low-, and middle-SDI regions had higher ASMR compared to high SDI regions. Notably, pulmonary hypertension (PH) is an insidious disease, and many patients are diagnosed and are referred to a specialist heart or lung center late in disease progression. The problem is exacerbated in low- and middle-income countries by difficulties in accessing right heart catheterization and therapeutic interventions (24). Consequently, equitable access to timely diagnosis and existing or emerging therapies are a critical priority event for PAH patients worldwide. Among the 21 GBD regions, we noted an increase in the case number and ASMR only in Central Asia, which alerted policymakers in the region to be highly critical and develop targeted intervention strategies. At the national level, the estimated ASMR varied significantly across 204 countries and territories, suggesting a strong heterogeneity in the burden of PAH worldwide. Importantly, the burden of PAH-related deaths suggests that different countries and territories should develop flexible public health policies to respond to the complexity of the situation.

Population growth and aging are important contributors to the increased mortality of PAH globally, regionally and nationally. According to the United Nations' World Population Prospects 2022, the world population is projected to reach 8.5 billion in 2030 and to increase further to 9.7 billion in 2050 and 10.4 billion by 2100, while the world's population is aging (25). Understandably, population growth and aging have inevitably driven the increasing mortality of PAH. In addition, PAH should be recognized as being caused by a combination of socioeconomic, racial, and ethnic disparities, lifestyle, and environmental exposure. Effective screening, early diagnosis, and adequate management, particularly equitable access to potentially life-saving treatments (26), are important in decreasing the mortality of patients with PAH.

This study performed a cross-national inequality analysis of PAH based on the standard health equity analysis methodology recommended by the World Health Organization and we observed a disproportionate increase in PAH mortality rates that should have been decreased in countries with high SDI. The anomalous association between PAH mortality and sociodemographic development may be caused by the following reasons. First, most countries with high SDI have more serious problems with smoking, particulate matter and household air pollution, hypertension, diabetes, and obesity, which inevitably contribute to increasing the mortality of PAH (27). In particular, although overall smoking prevalence has fallen in most high-SDI countries, secondhand smoke exposure and the rise of e-cigarette use continue to impart a measurable population-attributable risk for PAH (28, 29). Additionally, episodic but intense exposure to traffic-related PM2.5 and wildfire smoke in high-SDI settings has been linked to increased PAH hospitalisations and mortality (30, 31). The prevalence of hypertension, diabetes, and obesity has increased with socioeconomic development, and these are the main causes of PAH in high-income countries (32). In a large multicenter prospective cohort of patients with PAH, overweight patients and patients with obesity with PAH were associated with worse health-related quality of life. In addition, overweight patients and those with obesity had a trend toward increased incidence rates for hospitalizations when compared with normal-weight individuals (33). Second, most PH is related to underlying cardiopulmonary conditions, evidence suggests left-sided heart disease and lung disease are the most common causes of PH in economically developed countries (34), in recent decades, the increase in cardiopulmonary conditions may result in higher mortality rates of PAH in countries with high SDI. Additionally, we observed a significant increase in the SDI-related inequalities in PAH burden across countries over time, indicating that the investment in PAH diagnosis, management, and treatment may be insufficient as socio-demographic development occurs.

Projections with available data have demonstrated that the number of PAH cases will increase globally in the future due to population growth and continued aging. Population growth and aging have become a global phenomenon, which is a challenging issue for all countries of the world. Consequently, comprehensive strategies that include improved health management services for the elderly, prevention of risk factors at the primary care level, screening for PAH among the elderly and high-risk people, and access to high-quality medical services, contributes to the improvement of the disease burden associated with PAH.

Several limitations should be recognized in the present study. Firstly, the GBD study is heavily based on data collected, but the data sources are geographically limited, coming primarily from hospital-based registries and relying on historically different methods of classifying and identifying PAH. Additionally, right heart catheterization is necessary to diagnose PAH, but diagnostic rates can vary based on the level of development and level of medical care in different countries, and underdiagnosis may occur in countries with lower levels of medical care. As a result, the global burden of disease associated with PAH may be underestimated. Secondly, the PH is categorized into 5 clinical groups, due to the lack of relevant data from the GBD database, the disease burden and trends in other types of PH, such as PH due to left-sided heart disease, are not estimated in the present study, which restricts us from further extensive exploration. Thirdly, this study focused on analyzing mortality of PAH, but lacked data on the incidence, prevalence and disability-adjusted life years of PAH.

## Conclusion

In summary, PAH is a significant public health problem of global interest. The number of deaths has been increasing due to population growth and aging, but the ASMR has remained largely unchanged. Additionally, a strong heterogeneity in ASMR across 204 countries and territories. PAH burden is more pronounced in low-, and middle-SDI regions with poorly performing health systems. A significantly increased overall disease burden from PAH among females compared with males, suggests the need for sex-specific treatment strategies to enhance treatment benefits. Countries with higher sociodemographic development levels shouldered a disproportionately higher burden of PAH. The information provided in the current study can contribute to estimating the burden and trends of PAH and to developing more effective and targeted PAH health strategies.

## Data Availability

Publicly available datasets were analyzed in this study. This data can be found here: The Global Burden of Disease study 2021 is an open-access resource; data are available at https://vizhub.healthdata.org/gbd-results.
