# Supplementary material for: Mortality and trends of pulmonary arterial hypertension, 1990–2021: a population-based study
Source: Front Cardiovasc Med. 2025 Sep 3;12:1617610. doi: 10.3389/fcvm.2025.1617610 (PMC12440859; doi:10.3389/fcvm.2025.1617610)
Supplement: Supplementary file 1 [file Table1.docx]

**Table S1. The case number and age-standardized mortality rate of pulmonary arterial hypertension in 1990 and 2021, and its temporal trends from 1990 to 2021.**

| **Location** | **1990** | |  | **2021** | |  | **1990-2021**  **AAPC (95% UI)** |
| --- | --- | --- | --- | --- | --- | --- | --- |
|  | **Case number (95% UI)** | **ASMR (95% UI)** |  | **Case number (95% UI)** | **ASMR (95% UI)** |  |  |
| Afghanistan | 38 (19,61) | 0.41 (0.21,0.73) |  | 125 (56,189) | 0.67 (0.24,1.1) |  | 1.63 (1.47, 1.79) |
| Albania | 11 (8,15) | 0.52 (0.36,0.7) |  | 11 (7,19) | 0.28 (0.19,0.48) |  | -1.98 (-2.42, -1.54) |
| Algeria | 52 (34,94) | 0.31 (0.19,0.67) |  | 119 (43,185) | 0.40 (0.13,0.61) |  | 0.93 (0.55, 1.31) * |
| American Samoa | 0 (0,0) | 0.17 (0.12,0.39) |  | 0 (0,0) | 0.13 (0.07,0.28) |  | -0.85 (-0.89 - -0.82) |
| Andorra | 0 (0,0) | 0.25 (0.18,0.35) |  | 0 (0,0) | 0.11 (0.07,0.17) |  | -2.57 (-2.82, -2.33) |
| Angola | 18 (11,39) | 0.27 (0.13,0.56) |  | 30 (16,57) | 0.19 (0.09,0.36) |  | -1.09 (-1.32, -0.87) |
| Antigua and Barbuda | 0 (0,0) | 0.11 (0.1,0.12) |  | 0 (0,0) | 0.04 (0.04,0.04) |  | -3.20 (-4.02, -2.38) |
| Argentina | 137 (120,153) | 0.43 (0.38,0.48) |  | 108 (100,118) | 0.20 (0.19,0.22) |  | -2.48 (-2.71, -2.26) |
| Armenia | 7 (6,8) | 0.25 (0.21,0.31) |  | 3 (3,4) | 0.08 (0.06,0.09) |  | -3.83 (-4.38, -3.28) |
| Australia | 40 (34,52) | 0.22 (0.19,0.29) |  | 50 (42,57) | 0.12 (0.1,0.13) |  | -2.01 (-2.45, -1.57) |
| Austria | 20 (18,21) | 0.18 (0.16,0.19) |  | 25 (22,28) | 0.13 (0.12,0.14) |  | -1.05 (-1.53, -0.56) * |
| Azerbaijan | 37 (25,52) | 0.70 (0.48,0.99) |  | 54 (32,83) | 0.57 (0.36,0.85) |  | -0.69 (-0.99, -0.39) * |
| Bahamas | 2 (2,2) | 1.06 (0.93,1.18) |  | 2 (1,2) | 0.40 (0.32,0.5) |  | -3.02 (-3.41, -2.64) |

**Table S1. continued**

| Bahrain | 0 (0,1) | 0.19 (0.14,0.51) |  | 1 (1,2) | 0.16 (0.07,0.31) |  | -0.72 (-1.54, 0.10) |
| --- | --- | --- | --- | --- | --- | --- | --- |
| Bangladesh | 263 (124,437) | 0.43 (0.16,0.89) |  | 383 (189,664) | 0.30 (0.14,0.57) |  | -1.00 (-1.29, -0.71) |
| Barbados | 3 (3,3) | 1.06 (0.91,1.19) |  | 2 (1,2) | 0.36 (0.29,0.45) |  | -3.16 (-3.56, -2.76) |
| Belarus | 5 (4,6) | 0.04 (0.03,0.05) |  | 6 (5,7) | 0.04 (0.03,0.05) |  | -0.35 (-0.54, -0.16) * |
| Belgium | 44 (38,56) | 0.31 (0.27,0.39) |  | 45 (38,50) | 0.18 (0.16,0.2) |  | -1.79 (-1.91, -1.66) |
| Belize | 1 (1,1) | 0.46 (0.42,0.52) |  | 0 (0,1) | 0.15 (0.13,0.16) |  | -3.68 (-4.24, -3.13) |
| Benin | 7 (4,13) | 0.21 (0.08,0.43) |  | 13 (7,20) | 0.15 (0.06,0.27) |  | -1.05 (-1.18, -0.91) |
| Bermuda | 1 (1,1) | 1.73 (1.49,2.01) |  | 1 (1,1) | 0.57 (0.48,0.69) |  | -3.59 (-3.87, -3.31) |
| Bhutan | 1 (1,2) | 0.39 (0.17,0.67) |  | 2 (1,3) | 0.31 (0.15,0.52) |  | -0.76 (-0.90, -0.63) |
| Bolivia  (Plurinational State of) | 23 (12,37) | 0.42 (0.26,0.62) |  | 21 (15,30) | 0.24 (0.17,0.34) |  | -1.81 (-1.87, -1.75) |
| Bosnia and Herzegovina | 10 (7,14) | 0.27 (0.19,0.37) |  | 12 (9,16) | 0.21 (0.15,0.27) |  | -0.84 (-1.12, -0.55) |
| Botswana | 1 (1,2) | 0.18 (0.11,0.28) |  | 2 (1,2) | 0.10 (0.07,0.14) |  | -1.78 (-2.20, -1.36) |
| Brazil | 392 (371,410) | 0.38 (0.36,0.4) |  | 775 (710,818) | 0.33 (0.3,0.34) |  | -0.51 (-0.79, -0.24) * |
| Brunei Darussalam | 1 (0,1) | 0.48 (0.29,0.65) |  | 1 (1,1) | 0.27 (0.2,0.34) |  | -1.88 (-2.12, -1.63) |
| Bulgaria | 16 (13,18) | 0.16 (0.13,0.18) |  | 11 (8,15) | 0.09 (0.07,0.12) |  | -1.70 (-2.60, -0.80) * |

**Table S1. continued**

| Burkina Faso | 20 (9,38) | 0.32 (0.09,0.67) |  | 36 (17,59) | 0.27 (0.09,0.52) |  | -0.57 (-0.71, -0.43) |
| --- | --- | --- | --- | --- | --- | --- | --- |
| Burundi | 16 (9,31) | 0.37 (0.15,0.83) |  | 16 (6,32) | 0.21 (0.07,0.44) |  | -1.87 (-1.98, -1.75) |
| Cabo Verde | 1 (0,1) | 0.21 (0.06,0.48) |  | 1 (0,1) | 0.13 (0.05,0.24) |  | -1.53 (-1.81, -1.26) |
| Cambodia | 12 (6,26) | 0.16 (0.08,0.43) |  | 17 (10,38) | 0.13 (0.07,0.35) |  | -0.74 (-0.82, -0.66) |
| Cameroon | 15 (8,28) | 0.24 (0.09,0.48) |  | 32 (19,53) | 0.17 (0.08,0.31) |  | -1.15 (-1.30, -1.00) |
| Canada | 93 (85,100) | 0.31 (0.29,0.33) |  | 95 (83,104) | 0.14 (0.13,0.15) |  | -2.30 (-2.82, -1.78) |
| Central African Republic | 5 (3,11) | 0.3 (0.14,0.65) |  | 8 (4,14) | 0.25 (0.1,0.45) |  | -0.69 (-0.77, -0.61) |
| Chad | 11 (5,24) | 0.26 (0.07,0.64) |  | 27 (15,49) | 0.24 (0.08,0.47) |  | -0.13 (-0.31, 0.05) |
| Chile | 24 (23,26) | 0.22 (0.2,0.23) |  | 34 (30,37) | 0.14 (0.13,0.16) |  | -1.33 (-1.59, -1.07) |
| China | 4,059 (3,099,5,452) | 0.61 (0.46,0.83) |  | 7,318 (4,836,9,076) | 0.42 (0.28,0.51) |  | -1.26 (-1.53, -0.99) |
| Colombia | 34 (28,43) | 0.15 (0.12,0.19) |  | 52 (43,63) | 0.10 (0.08,0.12) |  | -1.44 (-1.75, -1.13) |
| Comoros | 1 (1,2) | 0.27 (0.12,0.5) |  | 1 (0,2) | 0.15 (0.07,0.3) |  | -1.95 (-2.72, -1.17) * |
| Congo | 3 (2,7) | 0.22 (0.13,0.45) |  | 4 (3,8) | 0.15 (0.08,0.29) |  | -1.28 (-1.45, -1.11) |
| Cook Islands | 0 (0,0) | 0.26 (0.19,0.54) |  | 0 (0,0) | 0.14 (0.08,0.33) |  | -2.03 (-2.17, -1.89) |
| Costa Rica | 8 (8,9) | 0.43 (0.39,0.48) |  | 6 (5,6) | 0.10 (0.09,0.12) |  | -4.59 (-5.15, -4.02) |

**Table S1. continued**

| Côte d'Ivoire | 6 (6,7) | 0.12 (0.1,0.14) |  | 4 (3,5) | 0.05 (0.04,0.05) |  | -0.99 (-1.24, -0.74) |
| --- | --- | --- | --- | --- | --- | --- | --- |
| Croatia | 11 (10,12) | 0.11 (0.1,0.12) |  | 9 (8,10) | 0.05 (0.04,0.06) |  | -3.10 (-3.71, -2.48) |
| Cuba | 13 (7,17) | 2.09 (1.09,2.75) |  | 15 (7,19) | 0.80 (0.38,1.02) |  | -2.14 (-2.44, -1.84) |
| Cyprus | 51 (43,60) | 0.39 (0.33,0.46) |  | 88 (76,101) | 0.43 (0.36,0.49) |  | -3.00 (-3.53, -2.48) |
| Czechia | 17 (10,30) | 0.24 (0.09,0.47) |  | 31 (16,50) | 0.18 (0.07,0.32) |  | 0.44 (-0.26, 1.14) |
| Democratic People's Republic of Korea | 43 (29,78) | 0.34 (0.22,0.65) |  | 95 (67,160) | 0.35 (0.24,0.58) |  | 0.07 (-0.01, 0.15) |
| Democratic Republic of the Congo | 58 (36,105) | 0.23 (0.1,0.46) |  | 86 (36,161) | 0.19 (0.07,0.39) |  | -0.61 (-0.70, -0.52) |
| Denmark | 17 (15,18) | 0.23 (0.21,0.25) |  | 21 (18,23) | 0.18 (0.15,0.19) |  | -0.88 (-1.32, -0.43) * |
| Djibouti | 1 (0,1) | 0.23 (0.11,0.49) |  | 1 (1,2) | 0.15 (0.07,0.29) |  | -1.24 (-1.57, -0.90) |
| Dominica | 0 (0,0) | 0.18 (0.11,0.32) |  | 0 (0,0) | 0.09 (0.07,0.23) |  | -2.01 (-2.19, -1.83) |
| Dominican Republic | 12 (9,16) | 0.17 (0.13,0.32) |  | 9 (6,23) | 0.08 (0.06,0.23) |  | -2.17 (-2.50, -1.83) |
| Ecuador | 18 (14,22) | 0.27 (0.2,0.34) |  | 27 (23,32) | 0.18 (0.15,0.21) |  | -1.38 (-1.73, -1.02) |
| Egypt | 727 (365,1,033) | 1.17 (0.77,1.55) |  | 282 (220,377) | 0.36 (0.28,0.5) |  | -3.72 (-4.02, -3.41) |
| El Salvador | 12 (9,16) | 0.29 (0.2,0.38) |  | 8 (5,16) | 0.12 (0.08,0.24) |  | -2.87 (-3.08, -2.66) |

**Table S1. continued**

| Equatorial Guinea | 1 (0,1) | 0.26 (0.12,0.55) |  | 1 (0,1) | 0.11 (0.06,0.2) |  | -2.63 (-2.92, -2.35) |
| --- | --- | --- | --- | --- | --- | --- | --- |
| Eritrea | 7 (4,14) | 0.30 (0.14,0.66) |  | 9 (4,16) | 0.21 (0.09,0.43) |  | -1.14 (-1.23, -1.04) |
| Estonia | 0 (0,0) | 0.02 (0.02,0.03) |  | 1 (1,1) | 0.04 (0.03,0.04) |  | 1.67 (-0.19, 3.55) |
| Eswatini | 1 (1,1) | 0.18 (0.12,0.29) |  | 1 (1,1) | 0.13 (0.09,0.18) |  | -1.03 (-1.13, -0.92) |
| Ethiopia | 92 (48,174) | 0.27 (0.1,0.54) |  | 97 (38,203) | 0.15 (0.05,0.3) |  | -1.93 (-2.01, -1.84) |
| Fiji | 1 (1,2) | 0.21 (0.13,0.44) |  | 1 (1,2) | 0.15 (0.08,0.32) |  | -1.05 (-1.13, -0.97) |
| Finland | 6 (5,7) | 0.09 (0.08,0.1) |  | 8 (7,9) | 0.06 (0.05,0.07) |  | -1.11 (-1.51, -0.71) |
| France | 239 (191,296) | 0.30 (0.24,0.37) |  | 325 (279,361) | 0.21 (0.19,0.23) |  | -1.15 (-1.36, -0.94) |
| Gabon | 1 (1,3) | 0.21 (0.1,0.45) |  | 1 (1,3) | 0.13 (0.07,0.24) |  | -1.50 (-1.55, -1.44) |
| Gambia | 1 (1,2) | 0.23 (0.07,0.46) |  | 3 (1,5) | 0.19 (0.07,0.34) |  | -0.73 (-1.18, -0.28) * |
| Georgia | 21 (16,27) | 0.37 (0.29,0.47) |  | 58 (45,73) | 1.01 (0.79,1.27) |  | 3.21 (2.45, 3.98) |
| Germany | 304 (254,350) | 0.27 (0.23,0.31) |  | 544 (453,604) | 0.26 (0.22,0.28) |  | -0.24 (-0.72, 0.24) |
| Ghana | 19 (11,37) | 0.22 (0.1,0.46) |  | 26 (14,44) | 0.13 (0.06,0.23) |  | -1.71 (-1.86, -1.55) |
| Greece | 32 (29,34) | 0.24 (0.23,0.26) |  | 87 (76,98) | 0.32 (0.29,0.36) |  | 0.92 (0.57, 1.27) |
| Greenland | 0 (0,0) | 0.77 (0.38,0.99) |  | 0 (0,0) | 0.19 (0.15,0.3) |  | -4.36 (-4.90, -3.82) |
| Grenada | 1 (1,1) | 0.81 (0.68,0.98) |  | 0 (0,0) | 0.26 (0.22,0.29) |  | -3.56 (-4.13, -2.98) |

**Table S1. continued**

| Guam | 0 (0,0) | 0.15 (0.1,0.35) |  | 0 (0,0) | 0.06 (0.03,0.12) |  | -3.26 (-3.83, -2.68) |
| --- | --- | --- | --- | --- | --- | --- | --- |
| Guatemala | 14 (12,16) | 0.26 (0.22,0.3) |  | 7 (6,8) | 0.06 (0.05,0.07) |  | -4.84 (-5.33, -4.35) |
| Guinea | 13 (7,27) | 0.26 (0.09,0.57) |  | 19 (9,32) | 0.21 (0.08,0.41) |  | -0.64 (-0.75, -0.53) |
| Guinea-Bissau | 2 (1,4) | 0.32 (0.13,0.6) |  | 3 (1,5) | 0.23 (0.1,0.42) |  | -1.05 (-1.16, -0.95) |
| Guyana | 1 (1,1) | 0.17 (0.15,0.19) |  | 3 (2,3) | 0.39 (0.3,0.5) |  | 2.88 (2.38, 3.38) |
| Haiti | 55 (21,95) | 0.87 (0.43,1.27) |  | 53 (27,87) | 0.49 (0.28,0.73) |  | -1.79 (-1.88, -1.71) |
| Honduras | 7 (4,10) | 0.26 (0.14,0.41) |  | 12 (8,18) | 0.20 (0.12,0.32) |  | -0.86 (-1.02, -0.70) |
| Hungary | 43 (38,49) | 0.32 (0.28,0.37) |  | 31 (25,38) | 0.16 (0.13,0.21) |  | -1.99 (-2.82, -1.16) * |
| Iceland | 0 (0,0) | 0.15 (0.14,0.17) |  | 1 (1,1) | 0.13 (0.11,0.15) |  | -0.64 (-1.06, -0.21) * |
| India | 1,756 (1,132,2,495) | 0.29 (0.17,0.46) |  | 2,612 (1,739,4,060) | 0.24 (0.15,0.39) |  | -0.63 (-1.00, -0.25) * |
| Indonesia | 185 (106,426) | 0.14 (0.08,0.42) |  | 268 (170,672) | 0.12 (0.07,0.37) |  | -0.52 (-0.59, -0.45) |
| Iran (Islamic Republic of) | 471 (307,593) | 1.40 (0.89,1.94) |  | 429 (286,494) | 0.61 (0.4,0.7) |  | -2.62 (-2.76, -2.48) |
| Iraq | 37 (21,57) | 0.24 (0.13,0.49) |  | 38 (20,80) | 0.15 (0.08,0.38) |  | -1.60 (-1.73, -1.47) |
| Ireland | 6 (5,6) | 0.15 (0.14,0.16) |  | 8 (7,9) | 0.11 (0.1,0.12) |  | -1.10 (-1.65, -0.54) * |
| Israel | 22 (19,29) | 0.48 (0.41,0.61) |  | 35 (30,39) | 0.27 (0.23,0.3) |  | -1.80 (-1.96, -1.63) |
| Italy | 171 (159,179) | 0.21 (0.2,0.23) |  | 138 (115,153) | 0.09 (0.08,0.1) |  | -2.84 (-3.12, -2.55) |

**Table S1. continued**

| Jamaica | 4 (4,5) | 0.20 (0.18,0.23) |  | 2 (2,3) | 0.07 (0.05,0.09) |  | -3.30 (-4.06, -2.53) |
| --- | --- | --- | --- | --- | --- | --- | --- |
| Japan | 385 (366,401) | 0.29 (0.28,0.3) |  | 1,002 (786,1,147) | 0.29 (0.25,0.32) |  | -0.05 (-0.37, 0.27) |
| Jordan | 2 (1,7) | 0.06 (0.03,0.29) |  | 4 (2,14) | 0.06 (0.02,0.2) |  | -0.07 (-0.42, 0.29) |
| Kazakhstan | 6 (5,7) | 0.05 (0.03,0.06) |  | 7 (6,9) | 0.05 (0.04,0.06) |  | 0.08 (-0.24, 0.40) |
| Kenya | 19 (10,40) | 0.14 (0.05,0.32) |  | 33 (14,65) | 0.12 (0.05,0.23) |  | -0.55 (-0.69, -0.42) |
| Kiribati | 0 (0,0) | 0.30 (0.17,0.68) |  | 0 (0,0) | 0.25 (0.16,0.57) |  | -0.57 (-0.62, -0.52) |
| Kuwait | 1 (1,1) | 0.07 (0.07,0.08) |  | 4 (3,5) | 0.14 (0.12,0.16) |  | 2.40 (0.11, 4.73) * |
| Kyrgyzstan | 3 (3,4) | 0.09 (0.08,0.11) |  | 2 (2,3) | 0.05 (0.04,0.06) |  | -2.16 (-2.89, -1.43) |
| Lao People's Democratic Republic | 8 (3,19) | 0.23 (0.11,0.54) |  | 9 (5,20) | 0.15 (0.09,0.36) |  | -1.30 (-1.39, -1.21) |
| Latvia | 1 (1,1) | 0.02 (0.02,0.03) |  | 4 (4,5) | 0.10 (0.09,0.12) |  | 4.71 (3.42, 6.01) |
| Lebanon | 21 (13,31) | 0.89 (0.55,1.34) |  | 23 (17,38) | 0.37 (0.28,0.59) |  | -2.79 (-3.03, -2.56) |
| Lesotho | 1 (1,2) | 0.14 (0.09,0.24) |  | 2 (1,3) | 0.14 (0.1,0.2) |  | 0.07 (-0.09, 0.23) |
| Liberia | 5 (3,10) | 0.27 (0.11,0.53) |  | 6 (3,11) | 0.19 (0.07,0.36) |  | -1.21 (-1.51, -0.91) |
| Libya | 15 (9,27) | 0.32 (0.2,0.64) |  | 18 (8,31) | 0.37 (0.17,0.64) |  | 0.50 (-0.24, 1.24) |
| Lithuania | 1 (1,2) | 0.04 (0.03,0.04) |  | 4 (4,5) | 0.08 (0.07,0.09) |  | 2.58 (0.61, 4.58) * |

**Table S1. continued**

| Luxembourg | 1 (1,1) | 0.27 (0.25,0.29) |  | 2 (2,3) | 0.21 (0.18,0.23) |  | -0.87 (-1.15, -0.59) |
| --- | --- | --- | --- | --- | --- | --- | --- |
| Madagascar | 38 (23,75) | 0.42 (0.2,0.8) |  | 58 (31,112) | 0.31 (0.15,0.61) |  | -0.94 (-1.00, -0.87) |
| Malawi | 17 (10,32) | 0.22 (0.1,0.43) |  | 19 (9,33) | 0.16 (0.07,0.3) |  | -0.87 (-0.99, -0.75) |
| Malaysia | 10 (7,30) | 0.08 (0.05,0.3) |  | 19 (11,65) | 0.07 (0.04,0.26) |  | -0.65 (-1.15, -0.14) * |
| Maldives | 1 (1,1) | 0.52 (0.37,0.69) |  | 1 (1,1) | 0.19 (0.15,0.3) |  | -3.25 (-3.38, -3.11) |
| Mali | 14 (8,30) | 0.23 (0.08,0.55) |  | 27 (13,53) | 0.18 (0.06,0.38) |  | -0.76 (-0.93, -0.60) |
| Malta | 0 (0,0) | 0.09 (0.08,0.1) |  | 1 (1,1) | 0.08 (0.07,0.1) |  | -0.38 (-0.77, 0.01) |
| Marshall Islands | 0 (0,0) | 0.33 (0.2,0.69) |  | 0 (0,0) | 0.23 (0.14,0.5) |  | -1.14 (-1.19, -1.08) |
| Mauritania | 3 (1,6) | 0.20 (0.09,0.46) |  | 4 (2,7) | 0.14 (0.06,0.28) |  | -1.15 (-1.48, -0.82) |
| Mauritius | 2 (2,2) | 0.26 (0.24,0.28) |  | 11 (10,12) | 0.71 (0.63,0.77) |  | 3.71(2.34, 5.09) |
| Mexico | 83 (75,100) | 0.15 (0.13,0.17) |  | 101 (89,112) | 0.09 (0.08,0.1) |  | -1.66 (-2.17, -1.15) |
| Micronesia (Federated States of) | 0 (0,0) | 0.36 (0.22,0.8) |  | 0 (0,0) | 0.24 (0.15,0.55) |  | -1.30 (-1.34, -1.25) |
| Monaco | 0 (0,0) | 0.18 (0.13,0.25) |  | 0 (0,0) | 0.15 (0.1,0.21) |  | -0.56 (-0.66, -0.46) |
| Mongolia | 20 (11,32) | 1.73 (0.99,2.86) |  | 34 (20,44) | 1.59 (0.91,2.05) |  | -0.35 (-0.67, -0.02) * |
| Montenegro | 0 (0,1) | 0.04 (0.02,0.24) |  | 0 (0,2) | 0.03 (0.02,0.2) |  | -0.77 (-1.07, -0.47) * |

**Table S1. continued**

| Morocco | 72 (40,126) | 0.33 (0.18,0.67) |  | 131 (50,201) | 0.43 (0.15,0.67) |  | 0.92 (0.78, 1.06) |
| --- | --- | --- | --- | --- | --- | --- | --- |
| Mozambique | 26 (14,47) | 0.27 (0.11,0.6) |  | 40 (18,70) | 0.24 (0.09,0.46) |  | -0.39 (-0.51, -0.27) |
| Myanmar | 70 (37,151) | 0.22 (0.12,0.55) |  | 75 (48,178) | 0.15 (0.1,0.42) |  | -1.14 (-1.22, -1.06) |
| Namibia | 1 (1,2) | 0.16 (0.11,0.23) |  | 2 (1,3) | 0.13 (0.09,0.19) |  | -0.60 (-0.71, -0.49) |
| Nauru | 0 (0,0) | 0.31 (0.2,0.63) |  | 0 (0,0) | 0.37 (0.24,1) |  | 0.49 (0.39, 0.59) |
| Nepal | 54 (31,85) | 0.38 (0.17,0.67) |  | 68 (34,117) | 0.31 (0.15,0.58) |  | -0.66 (-0.74, -0.59) |
| Netherlands | 30 (28,33) | 0.16 (0.15,0.17) |  | 48 (41,53) | 0.14 (0.12,0.15) |  | -0.53 (-0.90, -0.16) * |
| New Zealand | 5 (5,6) | 0.14 (0.13,0.15) |  | 8 (7,8) | 0.10 (0.09,0.11) |  | -0.93 (-2.08, 0.25) |
| Nicaragua | 4 (2,7) | 0.14 (0.07,0.3) |  | 2 (1,9) | 0.05 (0.03,0.19) |  | -3.51 (-3.73, -3.28) |
| Niger | 15 (7,27) | 0.28 (0.08,0.7) |  | 31 (11,61) | 0.23 (0.05,0.52) |  | -0.66 (-0.82, -0.50) |
| Nigeria | 161 (92,296) | 0.25 (0.09,0.52) |  | 228 (153,332) | 0.14 (0.07,0.23) |  | -1.79 (-1.91, -1.67) |
| Niue | 0 (0,0) | 0.26 (0.17,0.61) |  | 0 (0,0) | 0.23 (0.14,0.52) |  | -0.28 (-0.61, 0.05) |
| North Macedonia | 3 (2,4) | 0.15 (0.1,0.26) |  | 3 (2,6) | 0.12 (0.07,0.23) |  | -0.69 (-1.19, -0.18) * |
| Northern Mariana Islands | 0 (0,0) | 0.13 (0.08,0.3) |  | 0 (0,0) | 0.11 (0.07,0.28) |  | -0.56 (-0.70, -0.43) |
| Norway | 7 (7,8) | 0.14 (0.13,0.15) |  | 5 (4,5) | 0.05 (0.05,0.05) |  | -3.59 (-5.64, -1.49) * |
| Oman | 1 (1,4) | 0.11 (0.05,0.39) |  | 3 (1,5) | 0.11 (0.04,0.24) |  | 0.14 (-0.39, 0.67) |

**Table S1. continued**

| Pakistan | 311 (190,458) | 0.34 (0.18,0.55) |  | 485 (311,747) | 0.32 (0.18,0.56) |  | -0.20 (-0.35, -0.06) * |
| --- | --- | --- | --- | --- | --- | --- | --- |
| Palau | 0 (0,0) | 0.19 (0.11,0.42) |  | 0 (0,0) | 0.13 (0.06,0.3) |  | -1.21 (-1.29, -1.12) |
| Palestine | 4 (2,8) | 0.23 (0.1,0.59) |  | 4 (2,11) | 0.13 (0.05,0.41) |  | -1.86 (-2.02, -1.69) |
| Panama | 3 (3,4) | 0.19 (0.16,0.21) |  | 3 (2,3) | 0.06 (0.05,0.07) |  | -3.72 (-4.07, -3.36) |
| Papua New Guinea | 9 (5,14) | 0.3 (0.18,0.58) |  | 20 (13,36) | 0.27 (0.17,0.55) |  | -0.31 (-0.42, -0.21) |
| Paraguay | 2 (2,7) | 0.09 (0.05,0.27) |  | 4 (3,12) | 0.07 (0.04,0.22) |  | -0.54 (-0.77, -0.31) * |
| Peru | 42 (29,57) | 0.25 (0.19,0.32) |  | 43 (31,63) | 0.13 (0.09,0.18) |  | -2.06 (-2.92, -1.20) * |
| Philippines | 52 (34,115) | 0.11 (0.06,0.33) |  | 70 (48,184) | 0.08 (0.05,0.24) |  | -1.13 (-1.40, -0.85) |
| Poland | 69 (60,79) | 0.17 (0.14,0.19) |  | 77 (69,84) | 0.12 (0.11,0.13) |  | -1.14 (-1.48, -0.81) |
| Portugal | 28 (26,30) | 0.25 (0.23,0.26) |  | 56 (48,63) | 0.22 (0.19,0.24) |  | -0.56 (-1.20, 0.07) |
| Puerto Rico | 21 (19,23) | 0.60 (0.55,0.65) |  | 6 (5,7) | 0.10 (0.08,0.12) |  | -5.69 (-6.36, -5.02) |
| Qatar | 0 (0,1) | 0.17 (0.11,0.38) |  | 1 (1,2) | 0.13 (0.07,0.19) |  | -0.90 (-1.84, 0.05) |
| Republic of Korea | 42 (29,72) | 0.13 (0.09,0.27) |  | 39 (22,136) | 0.06 (0.03,0.17) |  | -2.67 (-2.89, -2.45) |
| Republic of Moldova | 0 (0,0) | 0 (0,0) |  | 0 (0,0) | 0.01 (0.01,0.01) |  | 3.96 (2.83, 5.10) |
| Romania | 120 (96,144) | 0.48 (0.39,0.57) |  | 170 (146,195) | 0.49 (0.42,0.55) |  | -0.04 (-0.39, 0.31) |
| Russian Federation | 515 (465,600) | 0.34 (0.31,0.39) |  | 221 (204,238) | 0.10 (0.1,0.11) |  | -3.70 (-4.43, -2.97) |

**Table S1. continued**

| Rwanda | 18 (11,39) | 0.35 (0.17,0.75) |  | 14 (6,30) | 0.17 (0.07,0.37) |  | -2.29 (-2.54, -2.03) |
| --- | --- | --- | --- | --- | --- | --- | --- |
| Saint Kitts and Nevis | 0 (0,0) | 0.21 (0.18,0.28) |  | 0 (0,0) | 0.07 (0.06,0.09) |  | -3.10 (-4.08, -2.11) |
| Saint Lucia | 1 (1,1) | 0.80 (0.72,0.89) |  | 1 (0,1) | 0.24 (0.2,0.29) |  | -3.81 (-4.31, -3.30) |
| Saint Vincent and the Grenadines | 0 (0,0) | 0.09 (0.08,0.1) |  | 0 (0,0) | 0.06 (0.05,0.07) |  | -1.12 (-1.57, -0.68) * |
| Samoa | 0 (0,1) | 0.27 (0.18,0.61) |  | 0 (0,1) | 0.20 (0.13,0.47) |  | -1.00 (-1.05, -0.96) |
| San Marino | 0 (0,0) | 0.10 (0.07,0.22) |  | 0 (0,0) | 0.05 (0.03,0.12) |  | -2.52 (-2.90, -2.12) |
| Sao Tome and Principe | 0 (0,0) | 0.16 (0.06,0.37) |  | 0 (0,0) | 0.13 (0.05,0.24) |  | -0.78 (-1.16, -0.39) * |
| Saudi Arabia | 14 (9,31) | 0.14 (0.08,0.39) |  | 18 (9,36) | 0.08 (0.04,0.2) |  | -1.79 (-2.18, -1.39) |
| Senegal | 13 (7,25) | 0.25 (0.09,0.49) |  | 18 (7,32) | 0.18 (0.06,0.33) |  | -1.02 (-1.20, -0.84) |
| Serbia | 13 (8,32) | 0.14 (0.09,0.39) |  | 15 (9,30) | 0.09 (0.06,0.19) |  | -1.37 (-1.60, -1.13) |
| Seychelles | 0 (0,0) | 0.12 (0.07,0.36) |  | 0 (0,0) | 0.08 (0.04,0.25) |  | -1.28 (-1.40, -1.16) |
| Sierra Leone | 10 (6,18) | 0.27 (0.12,0.49) |  | 12 (7,21) | 0.20 (0.08,0.34) |  | -1.01 (-1.17, -0.85) |
| Singapore | 6 (5,6) | 0.23 (0.21,0.24) |  | 6 (5,7) | 0.09 (0.08,0.1) |  | -3.01 (-3.83, -2.17) |
| Slovakia | 6 (4,12) | 0.10 (0.06,0.23) |  | 6 (3,11) | 0.06 (0.04,0.13) |  | -1.55 (-1.79, -1.31) |
| Slovenia | 1 (1,1) | 0.04 (0.03,0.04) |  | 2 (1,2) | 0.04 (0.03,0.04) |  | -0.12 (-0.79, 0.56) |

**Table S1. continued**

| Solomon Islands | 0 (0,1) | 0.26 (0.14,0.54) |  | 1 (0,1) | 0.22 (0.13,0.51) |  | -0.59 (-0.70, -0.48) |
| --- | --- | --- | --- | --- | --- | --- | --- |
| Somalia | 20 (8,37) | 0.36 (0.11,0.75) |  | 34 (9,73) | 0.27 (0.06,0.58) |  | -0.98 (-1.08, -0.88) |
| South Africa | 29 (24,37) | 0.11 (0.08,0.14) |  | 46 (35,55) | 0.09 (0.07,0.11) |  | -0.46 (-0.65, -0.28) * |
| South Sudan | 15 (8,29) | 0.32 (0.14,0.68) |  | 17 (9,30) | 0.24 (0.09,0.44) |  | -0.98 (-1.15, -0.81) |
| Spain | 131 (121,140) | 0.27 (0.25,0.29) |  | 241 (200,270) | 0.22 (0.19,0.24) |  | -0.67 (-1.00, -0.33) * |
| Sri Lanka | 55 (39,92) | 0.42 (0.32,0.7) |  | 101 (63,148) | 0.42 (0.27,0.6) |  | 0.11(-0.31, 0.54) |
| Sudan | 72 (37,132) | 0.37 (0.2,0.7) |  | 124 (60,183) | 0.42 (0.18,0.61) |  | 0.38 (0.23, 0.52) |
| Suriname | 2 (1,3) | 0.74 (0.49,0.9) |  | 2 (1,3) | 0.32 (0.24,0.47) |  | -2.61 (-2.93, -2.28) |
| Sweden | 17 (16,19) | 0.14 (0.13,0.15) |  | 26 (22,29) | 0.12 (0.11,0.14) |  | -0.52 (-1.49, 0.46) |
| Switzerland | 35 (30,43) | 0.36 (0.31,0.44) |  | 37 (31,42) | 0.19 (0.16,0.21) |  | -2.11 (-2.46, -1.76) |
| Syrian Arab Republic | 10 (5,38) | 0.09 (0.05,0.43) |  | 6 (3,33) | 0.06 (0.03,0.29) |  | -1.76(-2.14, -1.38) |
| Taiwan  (Province of China) | 13 (12,13) | 0.10 (0.09,0.1) |  | 77 (66,85) | 0.18 (0.16,0.2) |  | 1.99 (0.97, 3.02) * |
| Tajikistan | 41 (25,54) | 1.21 (0.74,1.66) |  | 53 (34,73) | 0.81 (0.53,1.09) |  | -1.34 (-1.76, -0.91) |
| Thailand | 40 (25,123) | 0.09 (0.06,0.38) |  | 62 (35,282) | 0.07 (0.04,0.28) |  | -0.94 (-1.28, -0.59) |
| Timor-Leste | 1 (1,3) | 0.19 (0.09,0.49) |  | 2 (1,4) | 0.15 (0.08,0.43) |  | -0.68 (-0.90, -0.47) |
| Togo | 5 (3,9) | 0.22 (0.09,0.45) |  | 9 (4,15) | 0.18 (0.07,0.31) |  | -0.70 (-0.88, -0.53) |

**Table S1. continued**

| Tokelau | 0 (0,0) | 0.29 (0.18,0.61) |  | 0 (0,0) | 0.29 (0.18,0.73) |  | 0.18 (-0.00, 0.36) |
| --- | --- | --- | --- | --- | --- | --- | --- |
| Tonga | 0 (0,0) | 0.20 (0.13,0.45) |  | 0 (0,0) | 0.14 (0.09,0.34) |  | -1.10 (-1.28, -0.92) |
| Trinidad and Tobago | 5 (4,5) | 0.47 (0.44,0.52) |  | 3 (2,4) | 0.18 (0.14,0.24) |  | -2.77 (-3.14, -2.40) |
| Tunisia | 15 (10,28) | 0.25 (0.16,0.54) |  | 40 (12,67) | 0.36 (0.11,0.6) |  | 1.07 (0.83, 1.31) |
| Turkey | 4 (3,5) | 0.18 (0.13,0.24) |  | 9 (7,12) | 0.23 (0.17,0.31) |  | -2.81 (-3.00, -2.62) |
| Turkmenistan | 0 (0,0) | 0.37 (0.22,0.77) |  | 0 (0,0) | 0.23 (0.15,0.52) |  | 0.75 (0.46, 1.04) |
| Tuvalu | 538 (331,724) | 1.20 (0.79,1.65) |  | 408 (313,562) | 0.50 (0.39,0.69) |  | -1.58 (-1.63, -1.53) |
| Uganda | 37 (20,68) | 0.28 (0.1,0.61) |  | 41 (22,72) | 0.15 (0.07,0.28) |  | -2.03 (-2.24, -1.81) |
| Ukraine | 41 (37,46) | 0.07 (0.06,0.07) |  | 42 (31,53) | 0.07 (0.05,0.09) |  | -0.01 (-1.23, 1.24) |
| United Arab Emirates | 6 (3,10) | 0.73 (0.38,1.09) |  | 10 (5,12) | 0.38 (0.2,0.53) |  | -2.09 (-4.39, 0.27) |
| United Kingdom | 106 (96,132) | 0.14 (0.13,0.18) |  | 119 (107,126) | 0.10 (0.1,0.11) |  | -1.10 (-1.37, -0.84) |
| United Republic of Tanzania | 47 (28,89) | 0.26 (0.11,0.52) |  | 62 (31,113) | 0.16 (0.07,0.31) |  | -1.51 (-1.68, -1.35) |
| United States of America | 971 (855,1,070) | 0.32 (0.28,0.35) |  | 1,785 (1,535,1,945) | 0.31 (0.27,0.33) |  | -0.09 (-0.30, 0.13) |
| United States Virgin Islands | 0 (0,0) | 0.42 (0.24,0.53) |  | 0 (0,0) | 0.16 (0.11,0.26) |  | -3.06 (-3.58, -2.54) |

**Table S1. continued**

| Uruguay | 8 (7,9) | 0.23 (0.2,0.25) |  | 8 (7,9) | 0.16 (0.14,0.17) |  | -1.30 (-1.49, -1.11) |
| --- | --- | --- | --- | --- | --- | --- | --- |
| Uzbekistan | 68 (54,85) | 0.46 (0.34,0.61) |  | 97 (77,119) | 0.37 (0.3,0.46) |  | -0.84 (-1.64, -0.04) * |
| Vanuatu | 0 (0,0) | 0.28 (0.17,0.57) |  | 0 (0,1) | 0.22 (0.14,0.52) |  | -0.70 (-0.87, -0.53) |
| Venezuela  (Bolivarian Republic of) | 14 (12,16) | 0.11 (0.09,0.12) |  | 12 (9,15) | 0.04 (0.03,0.06) |  | -2.57 (-3.08, -2.05) |
| Viet Nam | 70 (38,194) | 0.15 (0.07,0.5) |  | 106 (53,332) | 0.12 (0.06,0.4) |  | -0.78 (-0.82, -0.74) |
| Yemen | 44 (21,73) | 0.40 (0.16,0.81) |  | 106 (49,160) | 0.55 (0.21,0.9) |  | 0.99 (0.63, 1.34) |
| Zambia | 12 (7,25) | 0.24(0.12,0.49) |  | 26 (9,52) | 0.24 (0.08,0.49) |  | 0.11 (-0.01, 0.23) |
| Zimbabwe | 8 (4,14) | 0.15(0.08,0.25) |  | 18 (12,27) | 0.18 (0.12,0.26) |  | 0.66 (0.38, 0.94) * |

**Abbreviations:** ASMR, age-standardized mortality rate; UI, uncertainty interval; SDI, sociodemographic index; CI, confidence interval; AAPC, average annual percentage change.

*, indicates *P*-value less than 0.05.
