# Supplementary material for: Mortality and trends of pulmonary arterial hypertension, 1990–2021: a population-based study
Source: Front Cardiovasc Med. 2025 Sep 3;12:1617610. doi: 10.3389/fcvm.2025.1617610 (PMC12440859; doi:10.3389/fcvm.2025.1617610)
Supplement: Supplementary file 2 [file Table2.docx]

**Table S2. The predicted case number of mortality of pulmonary arterial hypertension to 2036 globally.**

| Year | Value | Type |
| --- | --- | --- |
| 1990 | 14,842 | Actual |
| 1991 | 14,994 | Actual |
| 1992 | 15,166 | Actual |
| 1993 | 15,365 | Actual |
| 1994 | 15,500 | Actual |
| 1995 | 15,680 | Actual |
| 1996 | 15,720 | Actual |
| 1997 | 15,766 | Actual |
| 1998 | 15,895 | Actual |
| 1999 | 16,173 | Actual |
| 2000 | 16,399 | Actual |
| 2001 | 16,603 | Actual |
| 2002 | 16,836 | Actual |
| 2003 | 17,084 | Actual |
| 2004 | 17,230 | Actual |
| 2005 | 17,568 | Actual |
| 2006 | 17,948 | Actual |
| 2007 | 18,577 | Actual |
| 2008 | 19,448 | Actual |
| 2009 | 20,315 | Actual |
| 2010 | 21,104 | Actual |
| 2011 | 21,516 | Actual |
| 2012 | 21,797 | Actual |
| 2013 | 22,018 | Actual |
| 2014 | 21,996 | Actual |
| 2015 | 21,934 | Actual |
| 2016 | 21,943 | Actual |
| 2017 | 21,843 | Actual |
| 2018 | 21,831 | Actual |
| 2019 | 21,961 | Actual |
| 2020 | 21,902 | Actual |
| 2021 | 22,021 | Actual |
| 2022 | 22,127 | Forecast |
| 2023 | 22,223 | Forecast |
| 2024 | 22,310 | Forecast |
| 2025 | 22,388 | Forecast |
| 2026 | 22,459 | Forecast |
| 2027 | 22,522 | Forecast |
| 2028 | 22,579 | Forecast |
| 2029 | 22,631 | Forecast |

**Table S2. continued**

| 2030 | 22,677 | Forecast |
| --- | --- | --- |
| 2031 | 22,719 | Forecast |
| 2032 | 22,756 | Forecast |
| 2033 | 22,790 | Forecast |
| 2034 | 22,821 | Forecast |
| 2035 | 22,848 | Forecast |
| 2036 | 22,873 | Forecast |
