# Supplementary material for: Mortality and trends of pulmonary arterial hypertension, 1990–2021: a population-based study
Source: Front Cardiovasc Med. 2025 Sep 3;12:1617610. doi: 10.3389/fcvm.2025.1617610 (PMC12440859; doi:10.3389/fcvm.2025.1617610)
Supplement: Supplementary file 3 [file Table3.docx]

**Table S3. The predicted age-standardized mortality rate of pulmonary arterial hypertension to 2036 globally.**

| Year | Value | Type |
| --- | --- | --- |
| 1990 | 0.352153793 | Actual |
| 1991 | 0.34999902 | Actual |
| 1992 | 0.348693946 | Actual |
| 1993 | 0.348136046 | Actual |
| 1994 | 0.346216421 | Actual |
| 1995 | 0.34531548 | Actual |
| 1996 | 0.341143611 | Actual |
| 1997 | 0.33679204 | Actual |
| 1998 | 0.334053484 | Actual |
| 1999 | 0.334085384 | Actual |
| 2000 | 0.332858534 | Actual |
| 2001 | 0.330818855 | Actual |
| 2002 | 0.328997257 | Actual |
| 2003 | 0.327425142 | Actual |
| 2004 | 0.323166913 | Actual |
| 2005 | 0.322006254 | Actual |
| 2006 | 0.321196972 | Actual |
| 2007 | 0.32475031 | Actual |
| 2008 | 0.332382243 | Actual |
| 2009 | 0.339176668 | Actual |
| 2010 | 0.344256579 | Actual |
| 2011 | 0.342443605 | Actual |
| 2012 | 0.338008772 | Actual |
| 2013 | 0.332953882 | Actual |
| 2014 | 0.324506554 | Actual |
| 2015 | 0.315620207 | Actual |
| 2016 | 0.307705916 | Actual |
| 2017 | 0.298657698 | Actual |
| 2018 | 0.291159904 | Actual |
| 2019 | 0.285703735 | Actual |
| 2020 | 0.278522901 | Actual |
| 2021 | 0.274415583 | Actual |
| 2022 | 0.271877251 | Forecast |
| 2023 | 0.270214307 | Forecast |
| 2024 | 0.269081364 | Forecast |
| 2025 | 0.268290568 | Forecast |
| 2026 | 0.267730665 | Forecast |
| 2027 | 0.267331001 | Forecast |
| 2028 | 0.267044412 | Forecast |
| 2029 | 0.266838385 | Forecast |

**Table S3. continued**

| 2030 | 0.266690066 | Forecast |
| --- | --- | --- |
| 2031 | 0.266583209 | Forecast |
| 2032 | 0.266506191 | Forecast |
| 2033 | 0.266450667 | Forecast |
| 2034 | 0.266410632 | Forecast |
| 2035 | 0.266381765 | Forecast |
| 2036 | 0.266360949 | Forecast |
